# Power dynamics and participation within humanitarian coordination groups: A case study of the MHPSS Taskforce in Lebanon

**DOI:** 10.1371/journal.pgph.0003041

**Published:** 2024-03-14

**Authors:** Michelle Lokot, Thurayya Zreik, Rozane El Masri, Sandy Chaar, Rayane Ali, Bassel Meksassi, Michele Kosremelli Asmar, Martin McKee, Bayard Roberts, Rabih El Chammay

**Affiliations:** 1 Health Services Research and Policy, London School of Hygiene and Tropical Medicine, London, United Kingdom; 2 Independent Consultant, Beirut, Lebanon; 3 Research and Development Department, War Child Holland, Beirut, Lebanon; 4 Higher Institute of Public Health, Saint Joseph University of Beirut, Beirut, Lebanon; 5 National Mental Health Programme, Ministry of Public Health, Beirut, Lebanon; Royal Infirmary of Edinburgh, UNITED KINGDOM

## Abstract

The humanitarian sector has often been criticised for its hierarchical power dynamics. Such dynamics often centre the priorities of ‘international’ actors, thereby marginalising the knowledge and expertise of those closest to the setting and play out in various fora, including coordination mechanisms. While guidance emphasises the importance of supporting local systems and government structures rather than creating parallel humanitarian structures, this approach is not consistently applied, creating challenges. We used a case study approach to explore how power relations influence the practice of the Mental Health and Psychosocial Support Taskforce in Lebanon, a nationally-led coordination mechanism chaired by the Ministry of Public Health with UN agencies as co-chairs. We conducted 34 semi-structured interviews with Taskforce members and other stakeholders coordinating with the Taskforce, including local non-governmental organisations (NGOs), international NGOs, United Nations agencies and government ministries. Interview transcripts were collaboratively analysed using Dedoose. We conducted feedback workshops with participants and integrated their feedback into analysis. We found that UN agencies and international NGOs are perceived as holding more decision-making power due to their access to funding and credibility—both shaped by the humanitarian system. Our findings also suggest that power dynamics arising mainly from differences in seniority, relations between ‘local’ and ‘expat’ staff, and language used in meetings may affect, to varying degrees, decision-making power and members’ voices. We also show how the agenda/focus of meetings, meeting format, language, and existing relationships with Taskforce leaders can influence levels of participation and decision-making in Taskforce meetings, ranging from lack of participation through being informed or consulted about decisions to decisions made in partnership. Our findings have broader implications for coordinating service delivery within the humanitarian sector, emphasising the need to reflect upon power imbalances critically and continually and to ensure a shared understanding of decision-making processes.

## Introduction

The humanitarian sector–which comprises varied international non-governmental organisations (NGOs), local NGOs, UN agencies, community-based actors and civil society more broadly—has often been critiqued for being bureaucratic, hierarchical, and lacking accountability to affected populations [1,2]. Fassin argues that inequality is embedded within the humanitarian sector, as it is within Western societies more generally [3], and proposes that a “hierarchy of humanity” drives the “asymmetry” between those who give and receive aid and between international/foreign actors and local/national actors [4]. His framing of power dynamics has been used to understand the power of international agencies within the humanitarian response, which often assumes that international actors alone hold technical knowledge [5,6]. This is manifest, for example, in how local staff often work on the frontline while expatriates hold management and decision-making roles [7]. Other asymmetries are apparent in seniority [8] and gender, with cultural and social norms placing women lower in organizational hierarchies [9]. Gender, race, nationality, and seniority intersect to exclude certain groups from decision-making [10]. In recent years, since the World Humanitarian Summit in 2016 in particular, discussions about power dynamics in the humanitarian sector have become more prescient as part of the push towards localising and decolonising humanitarian aid [11,12].

In this paper, we explore the power dynamics within a humanitarian coordination mechanism responsible for mental health and psychosocial support (MHPSS)–the MHPSS Taskforce in Lebanon. MHPSS is defined as “… any type of local or outside support that aims to protect or promote psychosocial well-being and/or prevent or treat mental disorders” [13]. People affected by forced displacement experience high levels of psychosocial distress, and while often overlooked in the past, MHPSS services are increasingly recognised as essential elements of the humanitarian response. Since 2005, the humanitarian response has been organised in clusters, such as health, protection, and shelter [14], but MHPSS is considered a cross-cutting issue that should be integrated across all clusters. It is recommended that MHPSS responses are coordinated in working groups or taskforces, which are intended to bring together relevant clusters and ensure MHPSS considerations and activities are incorporated at all levels and sectors. While this positioning is intentional, there has been a critique of this structure leading to a lack of dedicated focus on MHPSS, additional challenges in coordination, and side-lining of MHPSS in favour of other cluster priorities [15,16].

Our study focuses on power dynamics within the MHPSS Taskforce in Lebanon and responds to growing critiques of humanitarian power dynamics described above and the increased demand for decolonising the systems and structures that shape humanitarian responses. To date, as far as we know, this is among the first study to focus solely on power dynamics within a humanitarian coordination body. Thus, it helps fill a gap as there is currently a lack of clear guidance to inform best practice in decision-making within coordination groups in this sector, although recently published guidance from the UN Inter-Agency Standing Committee (IASC) on MHPSS coordination does stress the importance of government co-leading coordination as well as building consensus within coordination groups [15]. Specifically, there is a lack of empirical evidence and guidance on how humanitarian actors should consider power hierarchies that operate within coordination bodies, despite the cluster system and its working groups/taskforces being common to humanitarian responses. As such, this study represents a new effort to explore how the power hierarchies within coordination bodies, including those that are nationally led, may influence the effectiveness and inclusivity of MHPSS responses. In one published case study of the MHPSS Working Group responding to the Rohingya refugee crisis in Bangladesh, participants in the national working group were mostly senior staff without direct involvement in service provision, so information and decisions were not always well communicated to field staff and meetings were less informed by field realities [16]. The authors also noted that the group’s outputs tended to focus on developing national technical guidance documents, strengthening capacity, and conducting assessments, with local issues being neglected [16]. They described the need for MHPSS coordination groups to involve community-based actors who know the context best and to establish field-based coordination structures with clear reciprocal links to national efforts—either through national representatives attending field-based meetings or vice-versa. These examples illustrate how power dynamics inherent in the humanitarian sector, including within coordination bodies, might limit the extent to which local/field realities are considered. There is a clear need to explore further how power dynamics inherent in the humanitarian architecture might undermine efforts to decolonise and localise humanitarian responses and how best to mitigate this.

### Conceptualizing power

Several theories of power can be drawn on to understand power dynamics, including Lukes’ and Gramsci’s concepts of hegemony and “invisible power” [17,18], resistance and “hidden power” [19], the idea of “power/knowledge” as elaborated by Foucault [20], Bourdieu’s theory of “habitus” [21], and Gaventa’s “power cube” [22]. In this paper, we mainly draw on the last of these, which combines many of the dimensions and modalities of power captured in the frameworks above. Gaventa’s power cube considers different spaces, levels, and forms of power. ‘Spaces’ of power may be closed, limiting decision-making to certain actors who are usually power-holders; invited, where participants are actively encouraged to participate; or claimed or created, which refers to spaces organically created by those with less power. ‘Levels’ of power describes levels at which participation may vary—at the global, national, or local levels. ‘Forms’ of power include visible power, which is the most observable form of power and often exists within policy-making and other formal structures involved in governing decision-making; hidden power, which describes the power of certain groups or people to influence decision-making; and invisible power, which refers to the psychological and social factors, such as norms and beliefs, that may influence decision-making.

The power cube has been used to analyse power in many areas, including mental health, governance, and policy-making [23], revealing dynamics that shape hierarchies and participation that influence ‘power over’ (i.e. the ability to exert and accumulate more power) [24,25]. It also informs the choice of intervention points to achieve change, including how disempowered actors may negotiate their agency in decision-making or ‘power to’ [26].

We also draw on Arnstein’s “ladder of participation” [27], which provides a framework that places different forms of participation onto eight ‘rungs,’ or levels, of a metaphorical ladder corresponding to varying degrees of decision-making power, with the least participative at the bottom. Non-participative forms include manipulation and therapy. Token forms are where decisions are taken by those who hold power, but others are at least engaged in some parts of the decision-making process. They include information sharing, consultation, and placation. Finally, Arnstein describes three forms of full decision-making, or ‘citizen power:’ partnership, delegation, and citizen control. This enables the identification of “real power” in decision-making, as opposed to what Arnstein describes as “the empty ritual of participation”. The ladder of participation, originally conceived as a framework to conceptualise citizen participation in governance, planning, and programming, has also been applied widely in many sectors, including healthcare, social work, and environmental policy [28]. Arnstein’s model thus enhances our understanding of participation across the different spaces, levels, and forms of power described in Gaventa’s power cube.

### The MHPSS Taskforce in Lebanon

The MHPSS Taskforce, established in 2015, is the national coordination mechanism for relevant humanitarian responses in Lebanon. Its mission is “to ensure an effective, coordinated and focused inter-agency response to the MHPSS needs of persons living in Lebanon, with a special focus on persons affected by the Syrian crisis (displaced and Palestine Refugees from Syria as well as the most vulnerable within the Lebanese and Palestine Refugees in Lebanon host communities), in line with the national mental health strategy of Lebanon”. It is chaired by the National Mental Health Programme (NMHP) at the Ministry of Public Health (MOPH), with co-chairs from WHO and UNICEF. It comprises about 60 organisations (UN agencies, local and international non-governmental organisations, and ministries) working on MHPSS, with the aim of inviting organisations across all relevant sectors. These organisations meet regularly to coordinate activities, develop guidelines, and participate in the development and implementation of an annual action plan that guides the work of the Taskforce throughout the year [29]. At different points in time, working groups have formed within the Taskforce to implement the tasks in the action plan. There are multiple opportunities for members to be involved in making decisions within the Taskforce, such as during meetings, through providing feedback on documents, and through their activities in working groups. Before the COVID-19 pandemic, it held regional and national meetings, but only the latter are now held. The Taskforce does not receive direct funding.

The MHPSS Taskforce in Lebanon is the only nationally-led coordination mechanism in Lebanon, contrasting with coordination mechanisms in other humanitarian settings that international actors, such as UN agencies, may lead. It is an example of how aid is becoming more localised, aligning with recommendations at the World Humanitarian Summit in 2016 to shift leadership to national bodies and aligning with global guidelines on strengthening national systems rather than creating parallel systems of international actor-led responses. A 2016 performance evaluation of the Taskforce saw 25 Taskforce members reflecting on strengths and weaknesses and making recommendations to improve its operation. The main strengths identified in this internal document were coordination, collaboration, and communication. The main weaknesses were lack of commitment and engagement by Taskforce members, with members pointing to problems with meeting formats and language used for meetings.

This study was part of a broader case study of the government-led MHPSS Taskforce in Lebanon, within a research partnership (“GOAL”), bringing together academic and civil society organisations in Lebanon and the UK to strengthen mental health systems respond to the mental health needs of Syrian refugees and host communities in Lebanon [30]. This study looks specifically into how power dynamics influence the Taskforce’s work.

## Methods

We conducted semi-structured interviews with members of the MHPSS Taskforce and other Lebanese stakeholders between February and November 2021. We used Zoom due to the COVID-19 pandemic. We also collected background information from documents related to the Taskforce, specifically minutes from meetings and the 2016 evaluation.

While the interviews also examined barriers and enablers to the work of the Taskforce as part of the broader study, the material presented here focuses on power dynamics, responding to the overarching research question: how do power relations influence the practice of the MHPSS Taskforce in Lebanon? Topic guides (annexed) were informed by Gaventa’s power cube and Arnstein’s ladder of participation described above [22,23,27] and were developed in consultation with GOAL project partners, who included the National Mental Health Programme. We piloted the guides during workshops to ensure they fully reflected the issues being explored, that questions were appropriately phrased, and that English and Arabic translations were suitable.

Thirty-four (34) participants (28 women and 6 men) participated in semi-structured interviews. Participants were recruited using a list provided by the National Mental Health Programme. They were either members of the Taskforce (n = 30) or had worked closely with it (n = 4), representing a range of actors working on MHPSS in Lebanon. The gender composition of the participants reflects the fact that the majority of Taskforce members are women. They included local non-governmental organisations (NGOs) (n = 10) and international NGOs (n = 11), UN agencies (n = 8), governmental organizations such as the Ministry of Public Health (MoPH) and other ministries (n = 4) and consultants (n = 1). The majority of participants were Lebanese. We have labelled the quotes we use to designate the type of organisation of the participant only to preserve anonymity.

Interviews were conducted by the research team (made up primarily of TZ, RM, RA and BM, all based in Lebanon) in either English or Arabic, depending on the participant’s preference. They were audio recorded and transcribed verbatim into English by RM, RA, BM, other team members and external translators. Other research team members checked transcriptions for accuracy; ten per cent of the transcripts were back-translated to ensure the accuracy of the Arabic-English translation. Transcripts were then anonymised and uploaded into Dedoose, a cross-platform application for qualitative data analysis.

As part of our commitment to co-production, we adopted a collaborative analysis and writing process for this paper. The interviews were coded collaboratively in pairs by the research team (ML, TZ, RM, RA, BM, SC) based on a codebook and using the blind coding feature offered by Dedoose. We used deductive and inductive coding, meeting bilaterally in pairs and regularly in a group to discuss codes and share perspectives on the data. The process of collaborative coding is described in more detail elsewhere [31]. While Gaventa’s power cube and Arnstein’s ladder of participation influenced the development of topic guides, during analysis, we found that participant accounts closely matched Arnstein’s ladder but did not neatly map onto the power cube. As such, we adopted an inductive approach when analysing those parts of the data that were unrelated to participation. However, we draw on the power cube and other power theories where relevant in the Discussion section.

We also reflected on our positionality and power throughout the research process, including during analysis, as part of our approach to collaborative coding and analysis. All of our analysis team members were part of an international NGO in Lebanon, except for ML, who was part of an academic institution in the UK. As a team, we discussed how gender, education level, ethnicity, age, experience in the humanitarian sector and geographical location particularly affected how we engaged with the data as researchers and how we perceived participant reflections on power relations in the Taskforce. We also reflected on the complexities of researching a coordination group where leaders of the group were also involved in framing the study. Our study is also complicated by the positionalities of participants, whose views about power may be based on their own positioning in the humanitarian system, especially given the increased recognition of power hierarchies in the humanitarian sector in recent years [4–8].

We developed a list of key themes from the coded content from transcripts and used them to draft the paper in a small group involving ML, TZ, RM, SC, RA and BM. The initial themes were shared with small groups of 16 participants who agreed to join feedback sessions in August 2022. Two of the seven feedback sessions were held with the Taskforce leadership team. The feedback is reflected in the findings, demonstrating how interview participants were involved in the analysis process.

### Ethics

Ethical approval was obtained from Université Saint-Joseph de Beyrouth (Saint Joseph University of Beirut) (ref: USJ-2020-255, 21/01/2021) and the London School of Hygiene and Tropical Medicine in London (ref: 22793, 13/01/2021). We gave participants an Information Sheet covering the scope of the research study and the interviews before obtaining written informed consent. Participants’ names, titles and affiliations were anonymised in the transcripts and numerical codes were used.

## Findings

In seeking to understand how power relations may influence the practice of the MHPSS Taskforce in Lebanon, the following paragraphs outline the main themes related to power dynamics. We outline three key findings: 1) UN agencies and international NGOs are perceived to have more power and influence in decision-making in the Taskforce; 2) seniority, expatriate-local dynamics, language, gender, and the focus and format of meetings shape power dynamics to varying degrees, and thus influence voice and decision-making in Taskforce meetings; and 3) levels of participation of members in the Taskforce vary. In the first and second sections, we have presented findings based on the themes that emerged through our coding and analysis process rather than those based on existing theories of power for the reasons explained above. For the third finding on participation, we explicitly structure findings based on Arnstein’s ladder of participation as this framework aligned closely with the data emerging from participants.

### UN agencies and international NGOs are perceived to have more influence in the Taskforce as part of their power within the humanitarian sector

Many participants reported how UN and international NGOs have a stronger influence on decision-making than local/national NGOs and community-based organisations, even though a local government body chairs the Taskforce. Participants linked the influence of these actors to their power and position within the broader humanitarian sector. Participants sometimes grouped UN agencies and international NGOs together or referred to them interchangeably. Thus, one participant said, “UN agencies… kind of have a bit of weight, I would say, like in the Taskforce sometimes” (UN). In one feedback session, a participant reflected on how “UN organizations do have a bit more say, especially organizations who are sector chairs or co-chairs” and that “the more international organizations do have a bit more say”. However, this participant also commented, “I don’t feel like any decision has been made without—or, at least, most decisions are a bit inclusive and participatory, but maybe there are some improvements that can be taken”.

Participants linked the power of UN and international NGOs to the access to funding and the credibility that goes with being the UN or an international NGO. This reflects how power is distributed within the humanitarian system, as the UN and international NGO actors do not fund the Taskforce and thus lack financial power over it. One participant commented that the UN’s relatively high funding means that “they have more power to be engaged” in decision-making in the Taskforce, “because they will be the ones funding” MHPSS activities (NGO). Another participant discussed how UN agencies have “leverage” or “financial power”, adding: “when you have resources to implement a certain project, you have more power over other people who do not [have resources]” (UN). She commented, “of course this impacts decision-making” but noted how the Taskforce tried to align to the Mental Health strategy and government priorities. However, these dynamics do still “play a role” (UN). In one feedback session, a participant reflected how, referring to the UN: “[T]o be fair, there is a power, because the National Mental Health Programme of Lebanon, they don’t have the finances… So, the power of using politics because they [UN agencies] have the money, I can see the power”. However, in another feedback session, a participant reflected on how the influence of international actors was “not just because of the funding” but reflected their technical knowledge and production of research and other outputs.

Participants described how UN actors and international NGOs have influence within the Taskforce, making the link between power and credibility: “the bigger power dynamic goes to international NGOs, [and] especially UN agencies, and WHO. They are regarded always as the, the more credible” (NGO). For this participant, credibility reflects the way that the humanitarian system is structured: “[I]n general, in the humanitarian sector you feel it, you know. For example, international NGOs, they are regarded as more credible than local” (NGO). Another participant reflected on complaints from partners in the field about how “some NGOs are treated preferentially than others”, giving the example of local NGOs who they perceived might be more efficient or professional than some international ones, but who faced “very long discussions” for approvals for training or projects from the Taskforce, while for international actors’ approvals for training came through at “a fast pace” (UN). Participants noted that these dynamics may affect decision-making on the level of the Taskforce; however, according to feedback from Taskforce leaders, the length of discussions corresponds to the level of development of a specific project proposal rather than preference for international over local NGOs. Moreover, the Taskforce leaders clarified that the co-chairs, as well as the NMHP, actively support actors in developing their proposals.

Participants discussed how UN organizations were also helped by their habit of convening smaller, often bilateral, meetings from which others (such as local NGOs and community-based organisations) were excluded. As one participant commented: “[T]hey [Taskforce leadership] say that there are certain things that will be discussed later on (…) This means that they communicated with each other" (NGO). However, this was challenged in a feedback session with Taskforce leaders, where one participant argued that both local and international actors were involved in these meetings. The perception that a “core group”, comprising some members of the Taskforce leadership, is responsible for most decision-making in the Taskforce was articulated by many participants. Although Taskforce leadership clarified that no such formal group exists, unlike in other coordination groups, this perception remained prevalent among many participants, who suggested the need to diversify representation in the “core group”: “Maybe that might be an idea for the Taskforce if they want in the future to include in their smaller core group some representations from national and international NGOs, they can do it on rotation basis” (UN).

Participants also situated the findings about the influence of UN agencies and international NGOs within broader dynamics in the sector. In one feedback session, participants raised the issue of how people within the UN system are “trained to take power”. International actors were often described as being “hierarchical” in their way of working in interviews and feedback sessions, in that they acted in ways that were “dominant”: “if you’re not visible… you basically don’t have a career”. For example, one participant felt that this dynamic within international agencies meant that their staff were “not giving a lot of space to local or regional or national actors”. In light of these broader dynamics in the sector, participants suggested building bridges between local and international NGOs and revisiting the Taskforce processes to increase collaboration and allow more participation. One participant argued for a wider representation while another suggested that the influence of UN or international NGO actors was more about how individuals positioned themselves and perceived their own “importance”, suggesting the need for international NGOs to have more “modesty” in their own expertise and greater belief “in the local strengths and power”. They emphasised how the Taskforce leaders tried to ensure collaboration but suggested that some individuals may undermine these efforts by their behaviour.

In one feedback session, a participant contended that “having an understanding of how decisions get made in any structure anywhere is really important for participants”, arguing for greater clarity on decision-making within the Taskforce. In another feedback session, a participant considered that implementation processes were “less transparent” than planning decisions and called for more delegation of tasks such as developing guidelines and training content to those on the frontline who knew the issues best. Other participants suggested that there was delegation, but Taskforce members do not always have the time to follow through. In feedback sessions, however, the Taskforce leadership argued that: “maybe all these efforts [communication and collaboration on action plans] are not really seen… this is why maybe we’re seeing the lack of trust”. Participants stressed the value of the Taskforce being locally-led, emphasising how this model tackles many critiques of humanitarian responses: “We are the only coordination mechanism humanitarian that is chaired by the body from the Ministry… we don’t find [this] actually in other settings. More often than not the humanitarian system is a parallel system to the system in place and this is one of the weak, most, biggest weaknesses for the humanitarian system coordination…” (Government).

### Power dynamics and other factors that influence decision-making and voice in the Taskforce meetings

Many Taskforce members reported feeling comfortable when participating in Taskforce meetings, with genuine opportunities for participation: “There is always [the] possibility of providing feedback and agreeing and disagreeing (…). I personally haven’t felt that anyone was holding back” (Government). Another said: “This is the thing that we noticed so that there is complete comfort. There [is] smoothness, issues that are found anyone can talk about, anyone can give suggestions, even anyone can put complaints, criticisms; we do not call it criticisms” (NGO).

Participants did recognise factors influencing participation in decision-making, including power dynamics (such as seniority, local-expatriate, gender) and procedural factors, such as meeting format, language, agenda/focus of meetings, and established relationships with Taskforce leaders. Many of these factors interact, as discussed below.

#### Seniority

Participants had mixed perceptions of the effect of seniority (which here refers to being in more senior positions within organizational hierarchies) on participation in Taskforce meetings. Participants reflected on how senior actors needed to be present in Taskforce meetings for important decisions to be made: “when we ask MHPSS Taskforce partners to commit to a direction or to give a position on a decision, most likely they will have to go back to more senior people in their organizations more than just being able to direct reply” (Government). In contrast, more junior individuals tend to share experiences from the field.

MHPSS Taskforce members expressed the view that the Taskforce leadership supports the balanced involvement of both senior and junior members. However, they also emphasized how senior Taskforce actors have “more experience” and knowledge, and this may influence how much “space” they take in meetings: “it feels the senior people, they have much more space to talk, or they actually take themselves more space to talk” (NGO). A few participants referred to junior actors as “shy” in the presence of more senior ones. This affects their involvement in the meetings and their willingness to express ideas and thoughts: “if the organization sends a social worker or a therapist to attend, I feel they are shy, you know! (…) they are shy to share when they see there is a lot of coordination, when they see many people from high” (NGO).

#### Local-expatriate power dynamics

Participants had mixed perspectives on power dynamics between local (individuals who are Lebanese) and expatriate (individuals who are not Lebanese) Taskforce members. Some felt that the Taskforce leadership gives equal attention and recognition to everyone: “In general, I don’t think that the Taskforce like, you know, discriminate between local and expats voices. I don’t think that that occurs” (UN). However, multiple participants also shared a view that locals and expatriates occupied different positions in the power hierarchy, influencing how decisions are made and how comfortable members feel when participating in meetings. Some also mentioned how local/expatriate power dynamics intersect with seniority: “… usually when we have internationals attending, most of the time they are more senior positions [within their own organizations] than the locals attending” (Government).

Most people who discussed this topic explained that while it is important to benefit from expatriates’ international experience, it is crucial that they try to understand the context of the Lebanese situation: “they have to have a strong grasp of the context, regardless that they are international or no (…) to know well the context before representing their organization” (UN). However, some participants suggested that the assumption that expatriates don’t understand the setting might be present among Lebanese: “I feel that sometimes they [Lebanese actors] do undermine an expat’s opinion… they forget that expats can be experts in Lebanese context even if they weren’t living here their entire life” (NGO). Participants in the feedback sessions suggested that assuming expatriates have less knowledge may not be helpful for a setting like Lebanon, where some have been working for long times: “I don’t think that it’s necessarily always true to say, well, local folks are going to understand the context better or international folks are not going to understand the context. It’s often a continuum”.

The expatriate-local division was discussed further in other feedback sessions. One participant felt that the MHPSS Taskforce had less expatriate presence than other similar groups in Lebanon. However, she clarified how “the loudest voices are by Lebanese working for international organizations”, further nuancing the expatriate versus local power dynamic by suggesting that local actors within international organisations may have high influence.

#### Gender

Overall, MHPSS Taskforce members agreed that there are no gendered power hierarchies in the Taskforce: “I have witnessed the leadership style that is inclusive…I am absolutely feminist. So, I’m quite sensitive to moments when there is a lot of patriarchal behaviour; I haven’t witnessed that” (UN). The facilitation of the meetings was also described as inclusive: “I don’t personally think that gender is a variable in a sense where it is quite a mixed group. Also, the facilitation was also sometimes done by men, sometimes by women” (Government).

Most participants stressed how there is a higher representation of women within the Taskforce, and the large number of women was perceived to promote strong female participation: “All of them were women, 95% of the Taskforce were women (…) I didn’t feel there was any problem or any kind of gender dynamics” (Government). This was felt to be representative of women’s representation in the humanitarian sector more broadly. This was reinforced in the feedback sessions: “it’s because of the nature of the field in the humanitarian settings, mainly, there are more women than men”.

A few individuals went further, arguing that having gender non-conforming people attending meetings was a sign of the Taskforce’s inclusivity. One participant also noted how LGBTQ issues are discussed in meetings: “the fact that we’re targeting the mental health of all genders, also makes it more open space for everyone” (NGO).

#### Meeting format

Participants discussed how Taskforce meetings used to be held at the regional level, commenting on how the change to larger, centralized group meetings following the COVID-19 pandemic may have affected participation. In one feedback session, a participant argued that these were too big and “active participation is reduced” and that it is “always the same people like saying something and speaking up”. They suggested that returning to smaller regional meetings might enhance inclusivity and ensure that a broader group of people feel comfortable participating.

The format of meetings is also linked to language and availability of translation (discussed in the section below). Participants highlighted that before the pandemic, informal member-to-member translation occurred when Taskforce meetings were held in person, yet this is no longer happening with the virtual meetings.

#### Language

Language was described as influencing power dynamics and was linked to the power hierarchies between locals and expatriates. Since most Taskforce meetings are conducted in English, local actors may face challenges in expressing themselves: an NGO worker noted that “Sometimes you understand English but you are not being able to express yourself well in English”, and that while some local actors may “have the knowledge” and “know a lot” they “cannot express themselves in English”. Another participant suggested a translator would help “because not all the people that are in the field know English” (NGO). Another participant described the use of the English language as “very bad” and negatively impacting local actors who cannot share their concerns (UN).

There was some discussion of how using only Arabic would exclude expatriates; however, one participant argued that barely any expatriates attend Taskforce meetings, so the use of the English language is not justified. However, other participants felt that the sole use of English language is not a barrier to participation: “usually everybody speaks English, even a local organization they have the persons who have to coordinate with entities with donors etcetera, so usually even a local NGO they have English speaking coordinators. So usually it’s not something that is problematic” (Government) They suggested that members are welcome to speak in Arabic, and any member can translate to English for the others. In the feedback sessions, the importance of having meetings in Arabic with translation for English-speakers was discussed:

“[E]ven though it, of course, goes against my interests, but they should be in Arabic because I would say 80% of the Taskforce members are Arabic speakers… if you’re like very honest it would make the meeting so much more participatory, if they would just be done in Arabic language with maybe a translation for the English for the people like me who always speaking”.

Apart from the use of the English language, one participant mentioned how the use of technical, “medical” jargon (such as coverage for psychotropics) during meetings may also be challenging for some actors (NGO), as despite the Taskforce being a technical working group, some members may not have a strong clinical background. For example, one participant commented: “I didn’t understand the technical words because I’m not that much technical in the mental health part” (NGO). This comment from a local actor may be linked to this actor being more focused on community-level or psychosocial activities rather than specialised MHPSS services, which is also discussed in the section below. Other discussions with Taskforce leaders have also affirmed that this may explain this perception.

#### Agenda/Focus of meetings

Multiple participants expressed an over-focus on more specialised services within the taskforce, with insufficient focus on more community-level psychosocial services. One participant suggested that the role of the Ministry of Health in setting the agenda influences the disciplinary orientation of the Taskforce: “I think that other actors might feel that the MHPSS taskforce is not enough Psychosocial Support and too much Mental Health. Really that they focus a lot on the clinical side more than this other side of mental health (…), the taskforce focuses more on MH [Mental Health] maybe because of the interest of the ministry” (NGO). This seemed to intersect with power hierarchies by organisation type as well, since participants in interviews and feedback sessions discussed how within the Taskforce, local actors tended to focus on community-level activities. In contrast, international actors focused on more specialised or medical aspects. In the feedback sessions, one participant felt that there was a “tendency” in the Taskforce to report mental health rather than psychosocial indicators and that by focusing more on mental health, the Taskforce was “maybe not giving a voice to actors that are more on the PSS side”–who are often local actors—making PSS seem “less valuable”. Taskforce action plans and feedback from Taskforce leadership illustrate that in addition to the existence of a specialized PSS group with which the Taskforce coordinates, action plans focus on a range of needs including PSS, further nuancing this perception.

#### Established relationships

Participants discussed how different factors might influence the extent to which members can participate in the Taskforce. For example, the perception emerged that having established relationships with the Taskforce leaders may affect how active a member is within Taskforce and may also influence dynamics among members:

“I also feel that those people who are more active are those who are more dominant in terms of power dynamics if you want, the ones that are dominating the meetings are the ones who have personal working relationships with the heads”

(NGO).

This participant also suggested that those newer to the group may not be able to benefit from strong relationships with Taskforce: “they are quite confident to talk because they know what they are talking about, while others maybe do not have such strong working relationships or are new” (NGO). In a feedback session, one participant reflected that relationships with Taskforce leaders make it easier for members to participate: “People who know each other always feel more confident to speak up. I mean, like for me, personally, if I wouldn’t know, the Taskforce leaders, I would mainly not speak up in the Taskforce. So it’s really, really something about personal relationships”. Taskforce leaders emphasised that some people feeling more comfortable to talk because they have been in the group for longer may be simply a consequence of people knowing each other for a longer period, not necessarily reflecting stronger working relationships. Additionally, bilateral meetings are conducted with new actors to ensure they get briefed and up to speed.

### Varied forms of participation and decision-making power are evident within the Taskforce

Participants discussed participation in making decisions, focusing on attendance, development of guidelines and creation of annual action plans, as well as feedback on decisions related to the Taskforce itself. The different forms of participation in the Taskforce are mapped onto Arnstein’s ladder, with instances of no participation, being informed, consultation, and partnership.

#### No participation

Participants provided several examples that fit under the category of “no participation”, but these related to groups who are not members of the Taskforce rather than existing members not being provided with opportunities to participate or declining to do so. One such group were Syrian organisations, which might feel constrained from participating in a government-affiliated body because their employees are “volunteers” and not officially permitted to work under Lebanese labour law: “Syrian organizations are usually not participating because they are scared of the government proximity” (NGO). This participant felt this resulted in “more international or big organisations” being represented in the Taskforce than grassroots organisations.

Another example of a group not part of the Taskforce was MHPSS service users. One participant said that she was uncertain whether service users participated in developing Taskforce research and guidelines: “I would assume that they do ask for service users input but this is not something that is necessarily shared with the Taskforce” (NGO). During interviews and feedback sessions, leadership reflected on the challenges of increasing the participation of service users. In interviews, the ongoing creation of a new service user association was discussed as potentially creating opportunities for more meaningful engagement with this group.

The Taskforce leadership also reflected on the membership of the group, emphasising that one of the reasons that individuals or organisations might not directly be “invited” to participate is because the meetings are open to anybody: “[A]nyone, you can come as an individual clinician, if you are working within the humanitarian response, or as an association to express your willingness to be part of the taskforce, so we don’t extend invitations, so it’s an open meeting” (Government).

#### Being informed

During interviews, MHPSS Taskforce members shared examples that can be categorised as being informed, specifically receiving updates on decisions made by the Taskforce leadership and receiving meeting minutes. In these examples, participants emphasized the Taskforce’s role in information-sharing, however there was also some indication of feedback being sought prior to informing: “They do inform always everyone in all the updates and all the decisions that they are taking…” (NGO).

Several participants discussed how decisions about the action plan might be made by Taskforce leadership and then communicated to members during meetings for “validation”, arguing that this is not a meaningful opportunity to provide feedback. They reflected on how participation may be merely about being informed, especially at the end of a decision-making process, rather than having opportunities to input before a decision is finalised:

“I had a feeling… [it] was just for like validation and not really genuine validation, it’s like there are plans that are set and then they bring them to the Taskforce and they get discussed and then they move forward with them. So it’s more, this Taskforce is involved (…) at the end of the process and not like right at the beginning in the design (…) So the discussions maybe happened before in a kind core group meeting or something like that and then in the- this Taskforce is just to relay what happened and to validate”

(NGO).

“[S]ometimes you feel that it is done by only one party and other NGOs are only receiving information and not taking part (…) when we attend the meeting, it’s only to hear what they are doing alone”

(NGO).

During feedback sessions, Taskforce leaders had a different perspective–especially about the action planning process, describing it as “inclusive” and outlining a detailed process for making decisions based on consensus. However, one participant felt that the style of meetings had shifted over time. She said meetings were previously “more information sharing than active participation and coordination”. Still, she indicated that the leadership had “made a lot of efforts” to improve and meetings were “more active and participatory now” (NGO).

#### Consultation

Almost all examples of participation took the form of consultation. This included being asked to provide feedback on documents produced by the Taskforce leadership, as well as given opportunities to provide feedback on “challenges” and “opportunities” within the Taskforce. Many participants gave examples of being asked by leadership to provide input on drafts and action plans, with one member from the MoPH describing this process as “validat[ion]” (Government). Members also mentioned that they are often asked for feedback in different forms, including during meetings, by email, and through surveys and questionnaires.

Participants had mixed perceptions about the feedback process within the Taskforce. Most participants perceived that Taskforce leadership considers feedback and often implements changes accordingly: “they do listen to the feedback (…) and sometimes I can [give feedback], and based on my observation they do take it into consideration and work on accordingly” (NGO). Several members also spoke positively about the feedback process and described being actively encouraged to share feedback. Some participants also perceived that decisions are not solely taken by Taskforce leadership: “it’s not a top-down decision, it’s engaging all the actors to share their expertise in decisions so that together we can contribute to having a certain consensus” (NGO).

Several participants reported being excluded from the decision-making process despite being consulted for input and feedback. “Everything is decided, then they get back to us. They come back [for] feedback. Like, the decision-making, I don’t know who takes it, really” (NGO). A member of the Taskforce self-reflected on why this sentiment might be present, suggesting that the perception of some Taskforce members is that “the feedback that was given stops there, they are not really, if you want, heard (…) [and feel] as if nothing will change because we have a set strategy that will be implemented”. This participant felt that if feedback doesn’t align with what the Taskforce has in mind, it may not be incorporated (Government).

There were other perceptions of the feedback mechanism. Members described how feedback was not always clear or shared with the group, and some members expressed how they did not feel like their feedback was taken into consideration at all. One member mentioned that the process of decision-making is less participatory in cases where decisions need to be taken quickly: “Sometimes the documents are already developed, and things are already kind of done (…) I think sometimes this is needed when something has to come out quickly so it might not be as participatory, or as like, really inclusive" (UN).

#### Partnership

Although participants did not provide examples describing full decision-making–the highest level in Arnstein’s framework—two participants gave examples that can be considered to signify partnership in decision-making, which is the level below full participation. Both participants who mentioned partnership are members of the Taskforce leadership.

One participant described an example where some Taskforce members volunteered to produce documents and other material within working groups jointly:

“So that is a bit more in-depth involvement by [a number of] members who volunteered and then it is shared with the larger Taskforce to provide feedback so it’s nice to have a combination (…)”

(UN).

Another participant, referring to the same process, also mentioned the creation of technical committees “headed by different partners working [on] specific objectives of the action plan” (Government). Taskforce leadership mentioned other examples where opportunities to collaborate more intentionally were offered, observing that Taskforce members also have limited time and human resources to contribute to joint outputs.

## Discussion

Power dynamics are present in any decision-making or coordination mechanism but are especially important for the field of humanitarian aid, where power asymmetries often place “international” actors with funding in positions of influence. As outlined earlier in this paper, the cluster system and humanitarian architecture more broadly create several challenges for the humanitarian response, especially in a setting like Lebanon. In recent years, alongside the increased recognition of the need to localise and decolonise aid, there has been greater attention to the challenges of these power dynamics within the humanitarian sector. This study is among the first to rigorously explore how these power dynamics play out and influence practice within a coordinating body through a case study of the MHPSS Taskforce in Lebanon. The MHPSS Taskforce is a unique example in the humanitarian sector because it is the only nationally-led coordination group in Lebanon–representing a good example of efforts to localise. Our findings show that many of the power dynamics in this Taskforce reflect hierarchies inherent within the broader humanitarian system, with implications for similar coordination groups within and beyond Lebanon. We discuss the implications of each key finding below: the role of funding and power, the dynamics between international and local actors, the dynamics between senior and junior actors, the clinical versus PSS hierarchies, and participation and decision-making.

In our study, the perception of NGOs is that UN actors and international NGOs have greater voice and influence in decision-making within the Taskforce than national/local organisations was tied to their access to funding (e.g. unlike the NMHP which does not have funding) and credibility within the humanitarian system. This perception is not shared by the NMHP leadership in its relationships with the co-chairs. The relatively greater decision-making power of UN and international NGO actors than local actors reflects global levels of power in decision-making outside the state, as conceptualized in Gaventa’s power cube [22,23]. Barnett and Duvall’s taxonomy of power (developed for international relations but also applicable here) is also useful in framing funding and credibility as being structural–built into the system [32]. In reflecting on the implications of these findings, we also find Brandenburger & Nalebuff’s concept of “coopetition” important in thinking about how perceptions of power of different actors might be connected to competition and lack of trust in the humanitarian system. Coopetition describes the simultaneous presence of cooperation and competition [33]. In humanitarian emergencies, different organisations are required to develop trust with each other quickly for collaboration [34], while competing with each other for funding and attention [34,35]. Research in humanitarian settings has found both relationships of trust and mistrust among different organisations that are required to collaborate within a competitive environment [36].

Our finding about funding power aligns with existing critiques of the humanitarian funding structure that disproportionately directs funds towards international rather than local actors [37,38]. The unique aspect in Lebanon is that that the Taskforce is nationally-led, representing a new way of coordinating the humanitarian response. However, if local actors lack funding and human resources, as global literature suggests, they may have less influence on humanitarian decision-making. This is particularly relevant in the case of the Taskforce since lack of funding for the MHPSS Taskforce and action plans has been a major challenge. Despite this, international NGOs and UN agencies were still perceived to have more power in decision-making than other national organizations who are members of the Taskforce, even though the government, a local actor, is a co-chair. Despite the push towards localising funding in the broader humanitarian sector, this remains an area where little progress has been made [39], with implications not only for the MHPSS Taskforce but other decision-making bodies within the humanitarian system [38]. We also find more clarity may be required for Taskforce members on how the group operates and makes decisions to help tackle perceptions about decision-making processes within the Taskforce. Clearer communication may create opportunities for members to feel like their participation is more than being informed or consulted but includes opportunities higher on Arnstein’s ladder of participation, such as partnership [27].

The findings in the paper that junior actors tend to share field experiences, while senior actors are more engaged in decision-making on behalf of their organizations builds on existing studies [18] and has important implications for coordination in Lebanon and the humanitarian system. In the humanitarian system, decisions about who attends meetings occur at the organisational level and could be influenced by multiple factors, including politics and time availability. Additionally, the potential for hierarchies between local humanitarian workers and “expats”, which Pascucci found in her research in Lebanon and Jordan [7] and which has been found in other settings to privilege expatriate voices [10,38,40], adds another layer to the issue of seniority. Being in a more senior position of authority in an organisation and being an expatriate may provide additional privilege, potentially leading to local knowledge and expertise being undervalued in favour of outsider perspectives. However, our findings complicate existing studies on these power dynamics, as some expatriates have worked in Lebanon long enough to have an appropriate grasp of the context, resulting in a continuum rather than local and expatriate being set categories. Local-expatriate dynamics between individuals are also complicated by the NMHP (as chair of the Taskforce) being a local actor (a more unusual but recommended setup for coordination groups). Thus it is challenging to draw comparisons to other contexts, however the lack of funding power of the NMHP may lessen this influence. Reflecting on the findings about local-expatriate power dynamics, we also recognise that for the response to Syrian refugees, the actors are rarely local or from the same community (i.e. Syrian) but might most often be Lebanese. These nuances emphasise the importance of ensuring the participation of a range of voices–including Syrian service users—to mitigate these power hierarchies in decision-making. It is important that efforts to engage service users be meaningful and intentional, per Arnstein’s ladder of participation [27]. Embedded within the issues related to local-expatriate dynamics, our research also identified the power or “linguistic capital” [6] channelled through the use of the English language in meetings. We find Barnett and Duvall’s taxonomy of power helpful in framing the use of English as institutional power–part of how the humanitarian system operates [32]. Using the English language in humanitarian coordination meetings may exclude local actors [41,42]. Running Taskforce meetings in Arabic with English translation may represent an opportunity to shift power dynamics within the Taskforce, however this requires further discussion within the Taskforce. As these power dynamics stem from the overall structure of the humanitarian system, these findings have implications for other coordinating groups within the humanitarian system, specifically other clusters.

Our study also highlights opportunities to shift power through clearer communication about how decision-making occurs in the Taskforce. The perception that decisions are made by Taskforce leadership in a “closed” space through bilateral meetings and a “core group” may need to be corrected. However, drawing on Gaventa’s framework [23], “invited” spaces still exist to negotiate these decisions through information-sharing with members and member consultation on decisions and plans. Although some Taskforce members felt that final decisions occurred less transparently and that the use of feedback could be shared more clearly, the onus is not only on leadership but also on members to respond to requests for inputs and to contribute to tasks. More clarity may be needed for members on internal decision-making within the Taskforce.

In our study, the NMHP played an important role in influencing the Taskforce’s agenda, and as chairs, may hold what Gaventa’s power cube calls the most ‘visible’ power in public decision-making spaces, such as within Taskforce meetings [23]. This is not necessarily negative but has implications for who is perceived as holding authority. The discussion about the NMHP’s role included the perception among participants that the Taskforce emphasises clinical/medical aspects of mental health over more community-based psychosocial services, despite the emphasis on community-based services in Taskforce action plans. Psychosocial services were perceived as more of a focus for local and community actors, while mental health was seen as having a larger focus in international NGOs and UN organizations, perhaps reflecting the power dynamics discussed earlier. Psychosocial support services are also covered by the PSS Working Group, another body in the humanitarian coordination architecture in Lebanon, which may add to the confusion around the role of the Taskforce in this regard. This perception about the prioritisation of more medical or specialised services over psychosocial support in the Taskforce is different to the action plans and outputs of the Taskforce–indicating more work needs to be done with members to clarify the Taskforce position on this, given this has potential to create divisions in MHPSS coordinating groups as humanitarian actors may differ in their prioritisation of clinical or psychosocial services [43]. Despite MHPSS being intentionally integrated across multiple sectors globally in order to recognise the different levels and forms of support needed to adequately respond to the needs of the population, and despite the local mission of the Taskforce being to connect actors across sectors, the positioning of the coordination of the taskforce within the Ministry of Health may mean that extra efforts are needed to ensure that actors in non-health sectors feel equally engaged and supported. Miller and colleagues argue that clinical and psychosocial approaches are not inherently incompatible; instead, they address interconnected concerns and hold great potential for synergy when integrated into multilevel, multi-sectoral interventions [43]. We suggest that the Taskforce could engage in discussions with members to strengthen the harmonisation of these approaches.

Our participants emphasised how established relationships with Taskforce leaders may allow more confident engagement in the Taskforce. This aligns with the literature indicating that investing in high-quality relationships can contribute to better programme outcomes [44]. Such relationships with Taskforce leadership may make participation easier but can conversely make it challenging for newer actors. Staff turnover, a significant issue in humanitarian organisations [45,46], also poses challenges to efforts to influence and engage in bodies such as the MHPSS Taskforce. When staff holding strategic relationships move to other organisations or postings, the loss of institutional memory contained within these relationships may stall momentum.

### Limitations

Our study has several limitations. Firstly, following our co-production approach, the topic guide was developed with input from Taskforce leadership and the initial findings were discussed with Taskforce leadership. This helped ensure relevance, but could have influenced the findings given the leadership’s greater awareness of issues within the Taskforce. We sought to mitigate this by ensuring representation in the topic guide development and data analysis from the other GOAL partner organisations. The core analysis team also consisted of staff working for an international NGO and an international university rather than local organisations. The NMHP and other authors’ input helped ensure wider perspectives on the findings. Secondly, not all participants were available for interviews due to the COVID-19 pandemic, Beirut blast, and other economic and electricity challenges in Lebanon. A few participants had been less recently active in the Taskforce, so were less able to contribute on some current aspects of the Taskforce operations. We reflected on the participant’s level of engagement in the Taskforce during the analysis process to recognise the relevance of their reflections. Thirdly, while we tried to prioritise involving participants in the analysis process through feedback workshops, due to staff turnover within humanitarian organisations, their participation was not always possible. Lastly, because this study is among the first to explore power dynamics within a coordination body in a humanitarian response, our analysis is limited to participant interviews and feedback sessions only, rather than being able to compare with other settings or develop recommendations based on other contexts. There is a lack of other similar studies or guidance for decision-making in coordination groups to allow us to benchmark the Lebanon experience.

## Conclusion

This study highlights insights into how power dynamics influence decision-making in a humanitarian coordination body in Lebanon. Our findings suggest potential implications for how global humanitarian governance structures operate across multiple settings because the power associated with access to funding remains recurrent throughout the humanitarian sector and is not unique to the response in Lebanon. Despite efforts to localise humanitarian responses, funding remains disproportionately directed through international actors, which has ripple effects on power dynamics within the sector. Our study suggests a need to define how decision-making occurs within the Taskforce more clearly. As a nationally-led coordination body, the MHPSS Taskforce in Lebanon represents a new approach to humanitarian coordination. We also suggest that the MHPSS Taskforce should continue to be aware of power dynamics that endow senior or international actors with decision-making power and should intentionally seek to centre the perspective of junior and local actors who are often closest to the humanitarian response at the field level, for example through re-instating the regional Taskforce meetings. Balancing the need to hear from those most affected by issues–in this case, mental health service users—with the need to leverage decision-making authority is an ongoing challenge in the humanitarian sector. Engaging persons with lived experience in different modalities, including through the newly established Service User Association in Lebanon, may help ensure service users are consistently included. Many of the key issues identified in our study fundamentally stem from the structure of the humanitarian system, and further research on power dynamics (particularly within coordination groups) is required to set benchmarks for strengthening participation in decision-making and improving the effectiveness of MHPSS responses. Future research could also reflect on power dynamics more broadly in the humanitarian sector to contribute to ongoing momentum on the need to localize and decolonise aid.

## Supporting information

S1 Checklist (DOCX)
